# Experiences of Older Adults and Caregivers With Home Telemonitoring for Heart Failure in Canada: Qualitative Study

**DOI:** 10.2196/79797

**Published:** 2026-06-16

**Authors:** Guy Paré, Marie-Pierre Moreault, Philippe Voyer, Alexandre Castonguay, Marie-Soleil Hardy, Mickael Ringeval

**Affiliations:** 1 Department of Information Technologies HEC Montréal Montreal, QC Canada; 2 Faculty of Nursing Université Laval Québec, QC Canada; 3 Faculty of Nursing Université de Montréal Montreal, QC Canada; 4 Department of Computer Science Bentley University Waltham, MA United States

**Keywords:** digital health, heart failure, home telemonitoring, informal caregivers, older adults, patient experience, person-centered care, qualitative research

## Abstract

**Background:**

Home telemonitoring programs are increasingly used to support older adults living with chronic conditions such as heart failure (HF). While these interventions show promise for improving health outcomes and reducing care burden, their effectiveness depends largely on how patients and caregivers integrate digital technologies into everyday life and care relationships. However, relatively few studies have examined these experiences using conceptual frameworks that capture both functional and relational dimensions of care.

**Objective:**

This study aimed to explore the experiences of older adults and their informal caregivers participating in a home telemonitoring program for HF. Drawing on the Person-Based Approach and the Person-Centered Practice frameworks, we examined how participants engaged with both the technofunctional and relational aspects of the intervention.

**Methods:**

We conducted a qualitative study involving 34 patients, 28 informal caregivers, and 20 nurses across 3 primary care organizations in Quebec, Canada. The 6-month intervention included 4 connected devices used by patients (smartwatch, Bluetooth-enabled scale, voice-activated tablet, and a smart pill dispenser [xPill; Domedic]) and a mobile app for caregivers, complemented by remote nursing follow-up. Nurses reviewed patient data through a clinical dashboard at least once daily during weekday daytime shifts. Data were collected through semistructured interviews and field notes and analyzed using directed content analysis.

**Results:**

Participants’ experiences revealed both enabling and constraining factors across 2 key dimensions. Technofunctional engagement was shaped by digital literacy, emotional responses to the technology, alignment with daily routines, and access to technical or caregiver support. Relational aspects of care were influenced by perceived professional presence, opportunities for communication and shared decision-making, and the degree of emotional reassurance provided by remote monitoring. While many participants reported increased confidence and a sense of being supported, others experienced frustration, fatigue, or disengagement when the system disrupted routines or when feedback from clinicians was perceived as limited.

**Conclusions:**

Engagement with home telemonitoring technologies among older adults depends not only on usability but also on the relational context in which these technologies are embedded. Combining technofunctional and relational perspectives provides a more comprehensive understanding of how telemonitoring interventions are experienced and highlights the importance of personalized support, reliable technology, and sustained clinical engagement to promote meaningful adoption.

## Introduction

### Overview

Heart failure (HF) remains one of the leading causes of hospitalization and mortality among older adults globally [1,2]. As health care systems grapple with the dual pressures of aging populations and rising rates of chronic illness, the demand for scalable, patient-centered models of care has become increasingly urgent. Among the most promising innovations in this space are home telemonitoring programs, which aim to support early detection of clinical deterioration, foster self-management, and reduce reliance on in-person visits or rehospitalization [3,4].

Recent evidence supports the clinical value of these interventions. A systematic review and meta-analysis of mobile health technologies for home-based cardiac rehabilitation demonstrated significant improvements in patient outcomes and engagement, suggesting the potential of these tools to enhance chronic disease management at scale [5]. However, findings across studies remain mixed. The effectiveness of home telemonitoring programs often hinges on contextual factors such as system design, user characteristics, and the broader care environment [6]. This variability underscores the importance of understanding how these technologies are experienced and navigated by those who use them in their daily lives.

Older adults, in particular, represent a user group with unique needs and vulnerabilities when it comes to digital health technologies [7,8]. Differences in digital literacy, cognitive capacity, and physical function can pose challenges to sustained engagement. Emotional responses, such as anxiety, fatigue, or frustration, may also shape how older adults relate to technology over time. Moreover, their experience is often mediated by informal caregivers, whose involvement can critically influence the adoption, maintenance, and perceived usefulness of telemonitoring interventions. Yet, despite growing interest in user-centered evaluation, relatively few studies have provided in-depth, real-world accounts of how both patients and caregivers experience home telemonitoring programs.

This study seeks to address this gap by examining the lived experiences of older adults and their informal caregivers who participated in a home telemonitoring intervention [9] for HF across 3 primary care institutions in Quebec, Canada. In this study, we use the term home telemonitoring to refer to digital systems that collect and transmit patients’ physiological and self-reported health data from the home environment to clinicians for remote monitoring and follow-up.

The intervention involved 4 interconnected digital devices used by patients: a connected smartwatch, a Bluetooth-enabled scale, a voice-activated virtual assistant tablet, and a smart pill dispenser (xPill; Domedic). In addition, informal caregivers had access to the Proximity mobile app (Virtuose Technologies), which allowed them to receive alerts, monitor patient activity, and communicate with both patients and nurses. These digital tools were complemented by remote nursing follow-up, during which nurses reviewed patient data through a clinical dashboard, responded to alerts, and communicated with patients and caregivers when necessary.

Our objective was to understand how participants perceived and interacted with these technologies, how they navigated their technical and relational demands, and how the system was integrated into the routines and realities of everyday life. To achieve this goal, we draw on 2 complementary conceptual frameworks, namely, the Person-Based Approach (PBA) developed by Yardley et al [10] and the Person-Centered Practice (PCP) framework by McCance and McCormack [11]. The PBA provides a structured lens for understanding users’ cognitive, emotional, and behavioral engagement with digital health tools, emphasizing the importance of contextual relevance, emotional acceptability, and iterative user-centered adaptation. The PCP framework complements this by highlighting the relational and experiential dimensions of care, such as perceived professional presence, shared decision-making, and therapeutic alliance. By analyzing user experience through these dual lenses, this study offers a more nuanced understanding of the opportunities and limitations associated with home telemonitoring for older adults with HF. In doing so, it provides actionable insights for clinicians, designers, and managers aiming to improve the effectiveness, equity, and person-centeredness of these interventions. The following section introduces each framework in more detail and outlines its relevance to this study.

### Conceptual Background

#### The PBA Framework

To explore the techno-functional experience, we adopt the PBA to digital health intervention development [10]. The framework emphasizes the centrality of users’ lived experiences and psychosocial contexts in shaping how they interact with and respond to digital health tools. It is especially well suited to evaluating complex interventions such as multidevice telemonitoring programs, where sustained engagement is contingent not only on technical usability but also on the personal significance and practical fit of the technology in everyday life. Key elements of the PBA relevant to this study include the following: contextual tailoring, emotional and cognitive response to technology, perceived relevance and burden of technology use, and autonomy, control, and iterative engagement.

The PBA stresses the importance of designing or implementing interventions in ways that reflect the diversity of users’ real-life circumstances. This includes recognizing variation in cognitive functioning, literacy levels, physical capabilities, caregiving arrangements, and daily schedules. A telemonitoring system that is too rigid or uniform in its expectations may be experienced as alienating or unmanageable by users whose life contexts do not match the assumptions embedded in the system’s design.

The PBA underscores that user experience includes both affective and cognitive reactions. Feelings of reassurance, anxiety, confidence, or fatigue can influence whether and how users persist with an intervention. In the context of older adults, such responses are closely tied to familiarity with digital devices, past experiences with health care, and the presence or absence of technical or social support.

Perceived relevance and burden of technology use refers to how meaningful and manageable users perceive the intervention to be. For an older adult managing a chronic condition like HF, a telemonitoring device must clearly align with their health goals and offer value in their day-to-day life. At the same time, if the tool is perceived as cognitively demanding, repetitive, or disruptive to routines, it may generate a burden that outweighs perceived benefits, leading to frustration, disengagement, or early withdrawal.

Central to the PBA is the idea that users should feel a sense of agency when interacting with the intervention. Technologies that allow for flexibility and customization can reinforce users’ self-efficacy and support their identity as active participants in care. Conversely, systems perceived as overly prescriptive or difficult to modify may reassert feelings of passivity or dependence, particularly among older individuals already accustomed to clinician-led care.

In short, by applying the PBA, we aim to capture the nuanced ways in which older adults and their informal caregivers relate to the telemonitoring devices, how they negotiate its integration into their daily lives, and how usability, meaning, and burden are interpreted differently depending on their capabilities, routines, and support networks.

#### The PCP Framework

Originally developed to support quality improvement and professional reflection in nursing and allied health contexts, the PCP framework offers a comprehensive model of care that integrates professional ethos, care context, clinical process, and patient outcomes. It is especially relevant for understanding how care is experienced by patients in nontraditional or hybrid contexts, such as digitally mediated care environments, where therapeutic presence, trust, and relational continuity must be maintained at a distance. While the full framework includes 4 domains, namely, prerequisites, care environment, person-centered processes, and person-centered outcomes, this study focuses on the last 2, as these most directly pertain to the interpersonal and experiential dimensions of care in a home telemonitoring model.

Person-centered processes describe how health care professionals enact person-centered values in clinical practice. They represent the core relational and therapeutic mechanisms through which patients experience empathy, partnership, and a sense of agency in their care. One such process is sympathetic presence, which refers to the professional’s ability to be emotionally attuned and fully present with the patient. This presence is conveyed through attentive listening, sensitive responses, and compassionate behavior. In remote care settings, where nonverbal cues are absent, sympathetic presence must often be expressed through tone of voice, verbal phrasing, and consistent follow-up. Another key process is authentic engagement, which entails a genuine effort to understand the patient as a person, beyond their clinical profile or transmitted data. This means adapting interactions to align with each patient’s preferences, values, and life context, even when mediated by digital systems.

Shared decision-making is also central to PCP. It involves actively involving patients in decisions about their care by presenting options, seeking informed consent, and ensuring mutual understanding. In the context of home telemonitoring, this process often intersects with choices around the timing and content of reminders, the frequency of follow-ups, and the interpretation of patient-reported data. Finally, person-centered care involves working with patient beliefs and values, which requires acknowledging and respecting diverse worldviews, cultural backgrounds, and personal care goals, even when these may differ from standardized clinical routines or digital system defaults.

In a home telemonitoring context, these processes are especially vulnerable to being eroded or routinized, making their presence (or absence) highly meaningful to patients and caregivers.

Person-centered outcomes represent the experiential consequences of person-centered processes and serve as key indicators for evaluating the quality of care from the patient's perspective. One such outcome is the degree of involvement in care, which reflects whether patients feel that their voice is heard and that they have meaningful input into how care is delivered. In the context of home telemonitoring, this may involve being consulted about preferred methods and times for communication or having a say in how their personal health data are used. Another important outcome is the sense of well-being generated through care interactions. This encompasses emotional comfort, reduced anxiety, and a strengthened sense of self-efficacy. Expressions of reassurance, feelings of being understood, and the perception of being genuinely “cared for” at a distance are strong indicators of success in this domain. Finally, the development of a therapeutic alliance, a relationship characterized by trust, continuity, and mutual respect, remains fundamental even in digitally mediated care. Building this alliance requires proactive engagement from clinicians, timely responses to patient inputs, and communication practices that maintain the human element of care despite the technological interface.

By foregrounding these processes and outcomes, McCance and McCormack’s [11] framework helps us evaluate not just whether care is delivered, but how it is lived and interpreted by patients and informal caregivers. It provides a valuable counterbalance to the task-oriented lens of technology implementation by highlighting the relational texture of remote care.

## Methods

### Study Design and Setting

We adopted a qualitative study design guided by user-centered evaluation principles to explore the experiences of patients, informal caregivers, and nurses who participated in the home telemonitoring program. This approach emphasizes understanding how end users experience and engage with technology in the context of their everyday lives and care relationships [10,12]. It prioritizes the elicitation of first-hand accounts from those directly interacting with the intervention—namely, patients and caregivers—while also incorporating nurses’ perspectives to triangulate findings, enrich interpretation, and provide insight into how clinical support shaped user experiences.

Participants were recruited from 3 health care organizations in Quebec: Centre intégré de santé et de services sociaux (CISSS) Chaudière-Appalaches, CISSS Gaspésie, and CISSS Outaouais. These sites were selected by the project sponsor, the Quebec Ministry of Health and Social Services, to reflect the diversity and typical characteristics of primary care organizations across the province. Importantly, from the outset of the project, a formal governance structure was established to ensure coordination and collaboration among the 3 participating sites. Clinical managers responsible for the project in each organization worked closely together throughout its implementation, deployment, and follow-up. As such, the study was not designed to support cross-site comparisons. There was no a priori reason to expect meaningful differences in participant experiences between locations, and our analysis confirmed this assumption. Indeed, patients, informal caregivers, and nurses across sites reported broadly similar perceptions in relation to the 2 central dimensions of analysis, technofunctional use and relational care. Accordingly, findings are presented in an aggregated form.

### Intervention

The home telemonitoring intervention consisted of 4 interconnected digital devices installed in the residences of older adults diagnosed with HF. All participating patients were supplied with the same digital devices. The goal was to support daily self-monitoring of symptoms, vital signs, and medication adherence, while enabling remote follow-up by nurses. Each device was preconfigured to collect specific clinical data at defined frequencies, as follows:

- Patients interacted daily with a voice-activated tablet (Samsung [13]) that administered a structured symptom questionnaire (anamnesis) focused on HF warning signs and related physiological indicators. Items included: breathing difficulty, cough, dizziness, sleep disturbances, fatigue, retrosternal pain, digestion issues, urinary and bowel elimination, and disease-related anxiety. The system also issued prompts and reminders for weight measurement and medication intake.
- A smartwatch (Biobeat [14]) was used to continuously collect biometric data, including heart rate, blood pressure, and oxygen saturation, provided the patient wore it day and night. Data were passively captured and transmitted to the nurse monitoring platform.
- Patients were asked to weigh themselves using a Bluetooth-enabled weight scale (A&D Medical [15]) daily upon waking. No specific instructions were provided regarding attire during weighing (eg, nightwear vs daytime clothing), as the objective was to integrate the measurement into patients’ usual daily routines. The digital scale automatically transmitted weight data, which served as a proxy for fluid retention and clinical decompensation risk.
- A smart pill dispenser (xPill [16]) was used to issue audio-visual cues to prompt medication intake and record the date and time of each compartment opening to track adherence.

Each morning, patients received an audible alert from the voice-activated tablet signaling that it was time to complete a symptom questionnaire. They also had the option to initiate the questionnaire up to 1 hour before the scheduled alert. The questionnaire could be completed either by selecting responses directly on the tablet or by answering verbally. When responding orally, users were able to elaborate on their answers. In addition to completing the questionnaire, patients were expected to weigh themselves daily using the connected scale. The smart xPill automatically logged medication intake and allowed users to view as-needed prescriptions and detailed information about their medication regimen. The purpose of the xPill was to support medication adherence among patients and to enable nurses to monitor compliance remotely. Communication between the user and the nurse could be conducted through written messages or video calls initiated via the tablet, allowing for responsive and flexible clinical follow-up.

Together, these devices offered a comprehensive view of the patient’s evolving health status. Data were integrated into a secure clinical dashboard monitored by nurses, who could initiate follow-up interventions when anomalies or gaps in reporting were detected. Participating nurses were required to review their assigned patients’ data at least once daily during weekday daytime shifts. Although data were collected continuously (24 hours a day, 7 days a week), active monitoring occurred only during these daytime hours (8 AM to 4 PM), Monday through Friday. Outside of these hours, including evenings and weekends, patients experiencing health concerns were instructed to contact Quebec’s provincial health hotline (Info-Santé 811) or seek care at the nearest emergency department. Data collected overnight during the week were reviewed by nurses the following morning, while data from the weekend were examined on Monday morning.

The home telemonitoring program was designed as a 6-month intervention for participating patients. Patients who remained enrolled for the full duration used the monitoring devices continuously during this period. However, participation length varied for some individuals because several patients withdrew from the program after deployment of the devices. As reported in the Results section, the duration of participation among those who withdrew ranged from less than 1 week to approximately 7 weeks.

Informal caregivers were also integrated into the intervention through the Proximity mobile app. The app allowed them to view automated alerts designed to support the patient’s adherence to telemonitoring routines (eg, “your relative has not weighed themselves for 4 days,” or “the scale’s battery needs charging”) and to stay informed about the patient’s clinical status. Additionally, the app offered features for communicating via text message or secure videoconferencing with the patient and the assigned nurse.

### Participant Recruitment

A total of 20 nurses from the participating health care organizations were recruited by their supervisors. To participate in the project, they were required to complete an online training module on the clinical cardiac assessment of older adults [17]. This training consisted of 10 instructional videos, totaling approximately 4 hours, and concluded with a knowledge assessment.

Each nurse was responsible for identifying eligible patients from their caseload and initiating recruitment. Eligibility criteria for patients included the following: being aged 65 years or older, being francophone, having a diagnosis of HF, and currently receiving home care services related to that condition. These 4 criteria were mandatory. An additional, nonmandatory criterion was having an informal caregiver willing to participate in the study, aged 18 years or older, and possessing a smartphone or tablet with internet access.

**Figure 1:**
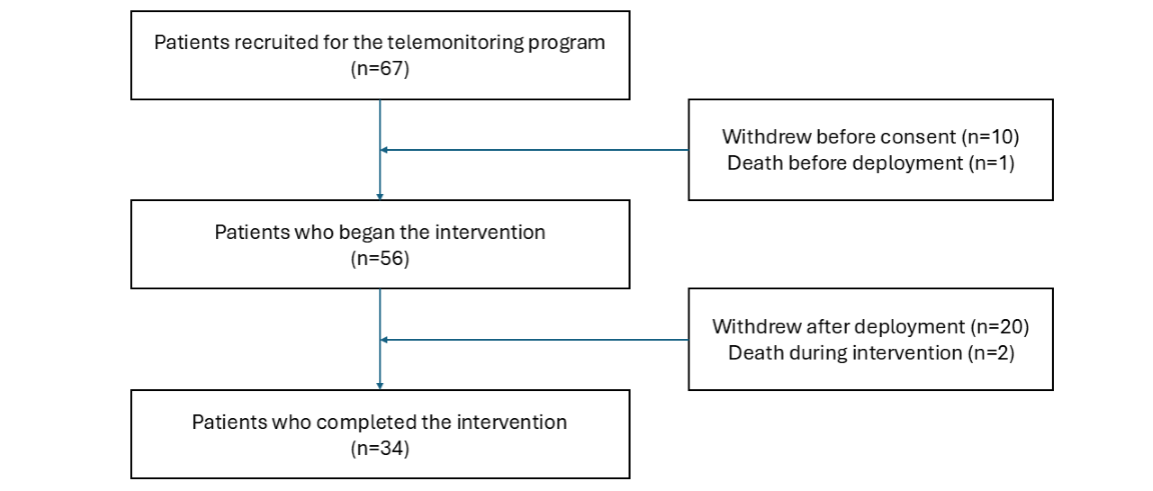
Participant flowchart in the home telemonitoring program.

A total of 67 patients were initially recruited into the study. Before the telemonitoring devices could be installed, however, 10 participants withdrew, while 1 patient died. For those who consented, the nurse proceeded to install the devices in the patient’s home and provided basic instructions on their use. Technical support throughout the study was provided by Virtuose Technologies [18], the project’s primary technology partner, alongside IT staff from each participating organization and the nurses involved in the intervention. During the intervention, 2 additional patients died, and 20 others chose to discontinue their participation. These withdrawals were observed across all participating sites. Among those who withdrew, the duration of participation varied widely, ranging from less than 1 week to more than 7 weeks. The reasons underlying early withdrawal are examined in the following section. Figure 1 summarizes patient recruitment, participation, and retention throughout the intervention.

Recruited patients were asked to provide the name and contact information of their informal caregiver, when applicable. This information was shared with both the research team and the primary technology provider. Caregivers were then contacted by a member of the research team, who explained the study procedures and obtained their informed consent. Among the 34 patients who completed the intervention, 28 were supported by an informal caregiver who also took part in the study. Once consent was obtained, caregivers received an email from Virtuose Technologies with instructions on how to download the Proximity mobile app on their smartphone or tablet.

Lastly, as part of the onboarding process, the patient’s community pharmacy was identified, and the pharmacy owner was invited to collaborate with the project to allow the prescription management system to interface with the smart xPill dispenser. Connection costs were fully covered by the company providing the smart xPill to enable seamless data integration.

### Data Collection

Semistructured interviews were conducted at the end of the study with all 34 patients who completed the intervention, 16 of the 20 patients who withdrew early, 21 informal caregivers, and 20 nurses. Among informal caregivers, 13 participated in individual telephone interviews, while 8 took part in joint interviews conducted together with the patient they supported. Among the nursing staff, 18 were interviewed face-to-face, and 2 participated via telephone.

Of the 34 patients who completed the program, 32 were interviewed in person in their homes, while the remaining 2 participated via telephone. All 16 patients who withdrew early were interviewed by telephone. These interviews were analyzed alongside those of participants who completed the intervention. Although generally shorter, they provided valuable insights into barriers to sustained engagement, including emotional fatigue, technical difficulties, and misalignment with daily routines. Consequently, they were particularly informative in identifying factors associated with disengagement and early withdrawal from the home telemonitoring program.

All interviews were conducted by a member of the research team with training in qualitative research and extensive prior experience studying digital health interventions. Conducting all interviews with the same interviewer helped ensure consistency in the interview process and familiarity with the study context. The interviewer had no prior clinical relationship with the participants.

Interview guides were developed by the research team to explore participants’ experiences with the intervention while remaining closely aligned with the study’s conceptual framing. Separate guides were prepared for patients, nurses, and informal caregivers to reflect their distinct roles in the program. The guides were informed by the 2 conceptual frameworks used in this study. While these frameworks helped structure the domains explored during interviews, the guides were designed to remain flexible and open-ended to allow participants to describe their experiences in their own terms and to surface issues not anticipated by the frameworks. The interview guides are provided in Multimedia Appendix 1.

No repeat interviews were conducted. However, the combination of interviews across multiple participant groups and iterative analytic discussions allowed the research team to refine and validate emerging interpretations.

The average duration of interviews varied by participant type: 45 to 60 minutes for patients who completed the program, 20 to 30 minutes for nurses, and 5 to 10 minutes for patients who withdrew early and informal caregivers. All interviews conducted in person were audio-recorded with participants’ consent, except for 2 patients who declined recording. Telephone interviews were not recorded, but detailed notes were taken immediately after each interview to capture key points, striking statements, and unexpected reactions. Audio recordings were listened to in their entirety by the interviewer and the lead author to ensure familiarity with the full content of each interview.

Rather than producing full verbatim transcripts for all interviews, segments of the recordings directly relevant to the study’s research objective were transcribed verbatim. These excerpts included both confirmatory and disconfirming observations related to participants’ experiences with the home telemonitoring intervention. Field notes and analytic discussions among all team members were used to ensure that themes emerging outside the conceptual frameworks were not overlooked during the coding process.

### Data Analysis

We conducted a directed content analysis, following the approach outlined by Hsieh and Shannon [19]. This method enabled the systematic examination of qualitative data using existing theoretical frameworks to guide the initial coding process while remaining open to insights emerging from participants’ accounts. Interview notes and transcripts were first reviewed in their entirety to foster immersion in the data and develop a comprehensive understanding of participants’ experiences.

A preliminary coding scheme was developed based on the study’s main objective and sensitizing concepts drawn from the 2 guiding frameworks: the PBA [10] and the PCP framework [11]. The 2 frameworks were applied in a complementary manner rather than as independent analytic lenses. During coding and interpretation, particular attention was given to how participants’ technofunctional experiences with the devices interacted with their relational experiences of care. For example, technical frustrations or usability challenges sometimes influenced perceptions of clinical support, whereas timely and empathetic follow-up from nurses often mitigated negative reactions to the technology. This integrative perspective helped illuminate how digital tools and relational care processes jointly shaped participants’ overall experience of telemonitoring.

The interviewer also led the coding process and was therefore highly familiar with the interview material and study context. The coding process was highly iterative. Interview notes and transcripts were reviewed to identify segments relevant to the study’s objective, which were then coded using sensitizing concepts derived from the 2 frameworks. Throughout the analytic process, coding decisions and emerging interpretations were discussed during regular team meetings. These discussions allowed the research team to examine the coder’s analytic reasoning, question assumptions, and refine the organization of themes. Differences in interpretation were addressed through discussion until a shared understanding of the coding structure was reached.

Although the analysis was initially guided by the 2 conceptual frameworks, attention was paid to identifying experiential elements that did not fit neatly within these categories. The coding process, therefore, remained flexible and iterative, allowing the coding scheme to evolve as new insights emerged from the data.

### Rigor and Reflexivity

Two strategies were used to enhance the trustworthiness of the analysis: triangulation of perspectives and collaborative interpretation. Specifically, we triangulated insights across the 3 participant groups interviewed (patients, informal caregivers, and nurses) to capture multiple perspectives on the home telemonitoring experience. Reflexivity was supported through regular analytic discussions among team members with diverse disciplinary backgrounds, including health informatics, nursing, and qualitative research.

We also drew on member-checking opportunities during follow-up meetings with clinical managers from each participating organization, during which preliminary interpretations were discussed and validated in light of their knowledge of the program’s implementation and patient population.

Analytic transparency was supported by providing a detailed description of the coding procedures, explaining how the conceptual frameworks guided the analysis, and illustrating themes with representative excerpts from participants across the 3 groups interviewed. Data coding and theme development were conducted primarily by 1 member of the research team, with iterative peer debriefing sessions held with other team members throughout the analytic process. These discussions helped challenge preliminary interpretations, ensure that coding decisions remained grounded in participants’ accounts, and refine the organization of themes. Particular attention was given to identifying experiences that diverged from dominant patterns in the data, such as frustration, fatigue, or disengagement with the technology.

Although the research team was not involved in the initial design of the telemonitoring intervention, 2 authors participated in monthly meetings of the project’s executive committee, and 1 author regularly attended weekly operational meetings in each participating organization. While their role in these settings was primarily observational and advisory, we acknowledge that this presence, and occasional input, may have influenced implementation dynamics or participant behavior. To mitigate this potential bias, we relied on triangulation of data sources, consensus-based interpretation, and a sustained effort to ground our analysis in participants’ experiences rather than in project objectives or institutional priorities.

### Ethical Considerations

This study received ethics approval from the ethics board of the CIUSSS Chaudière-Appalaches (#MP-23-2023-1037) on July 11, 2023. Ethical approval for the 2 other participating sites (CISSS Outaouais and CISSS Gaspésie) was granted through institutional agreements recognizing the lead site’s ethics review. As noted above, all participants received information sheets and provided written or verbal informed consent prior to participation. Identifying information was removed from notes and transcripts, and data were stored on secure, password-protected servers. Participation in this study was voluntary and uncompensated.

As part of the informed consent process, patients were asked to identify the informal caregiver they wished to involve in the intervention. Only the caregiver explicitly authorized by the patient was granted access to the patient’s health-related data via the Proximity mobile app. Patients were informed about the nature of the data shared, and participation remained entirely voluntary. For participants with limited digital literacy, additional support was provided to ensure they understood the implications of data sharing and retained autonomy in decision-making.

## Results

### Profile Participants

As shown in Table 1, 44% (15/34) of the patients who completed the intervention were female, with a mean age of 83 (SD 7.7) years. A total of 38% (13/34) resided in private seniors’ residences, and more than half lived with a spouse or one of their children. Most participants (28/34, 82%) received active support from an informal caregiver as part of the project. In most cases, the caregiver was one of the user’s children, followed by spouses and members of the extended family. In terms of perceived health status, 47% (16/34) of users described their health as fair, while the others considered it to be good or very good.

**Table 1:** Profile of the patients who completed the intervention (n=34).

Characteristic		Value
Female, n (%)		15 (44)
Age (years), mean (SD)		83 (7.7)
Reside in private seniors’ residences, n (%)		13 (38)
Live with a spouse or relative, n (%)		21 (62)
Have an informal caregiver, n (%)		28 (82)
**Perceive health status as, n (%)**		
	Fair	16 (47)
	Good or very good	18 (53)
**Perceive their level of digital literacy as, n (%)**		
	Low or very low	18 (53)
	Average	9 (26)
	High or very high	7 (21)
**Use of digital devices, n (%)**		
	Smartwatch	34 (100)
	Virtual assistant tablet	34 (100)
	Bluetooth-enabled scale	31 (91)
	Smart xPill dispenser	21 (62)
**Perceive their autonomy level in the use of digital devices as, n (%)**		
	Full autonomy	27 (79)
	Used with help of caregiver	4 (12)
	Mainly used by caregiver	3 (9)

Regarding digital literacy, more than half of the participants reported having low or very low comfort with digital technologies. In this context, approximately 20% (7/34) were assisted by their informal caregiver in completing the symptom questionnaire (anamnesis) and logging their daily medication intake using the smart xPill dispenser. All patients used the connected smartwatch, and the vast majority also used the connected scale. However, about one-third of participants were unable to use the smart xPill, as their community pharmacy was not yet connected to the system.

Among the individuals who initially agreed to participate but ultimately did not sign the consent form, several expressed concerns about their ability to manage the technological devices during formal follow-up. Some described their health as too fragile to take on the additional demands of the project and expressed fears that participation might cause further stress or anxiety. Others felt overwhelmed after reviewing the information sheet or watching the onboarding videos, which led to doubts about their ability to manage the devices. In some cases, these concerns were discussed with informal caregivers, resulting in a mutual decision to decline participation. Overall, these early withdrawals appear to have been driven by emotional apprehension, perceived cognitive or physical limitations, and a subjective sense of unpreparedness to engage with digital health technologies.

Another group consisted of patients who withdrew after the telemonitoring devices had been installed in their homes. This group included a higher proportion of women (12/20, 60%) and had a slightly higher mean age of 84 (SD 6.9) years. As shown in Table 2, 60% (12/20) of these participants had an informal caregiver involved in the project. On average, they remained enrolled for 4.6 weeks before deciding to discontinue their participation. Compared with the 34 patients who completed the intervention, the postdeployment withdrawal group tended to be older and had lower rates of caregiver involvement.

**Table 2:** Demographic characteristics of patient subgroups by participation status.

Characteristic	Surviving patients who withdrew early after deployment (n=20)	Patients who completed the 6-month intervention (n=34)
Female, n (%)	12 (60)	15 (44)
Age (years), mean (SD)	84 (6.9)	83 (7.7)
With an informal caregiver, n (%)	12 (60)	28 (82)
Duration of participation (weeks), mean (SD)	4.6 (1.1)	24 (0.2)

Interviews with the 20 patients who withdrew after deployment revealed a distinct set of challenges arising from their direct experience with the system. Many reported emotional fatigue from repetitive tasks, cognitive overload due to the use of multiple devices, and frustration with technical issues such as unreliable connectivity or unintuitive interfaces. Some also found it difficult to adjust their daily routines to accommodate the system’s requirements, such as scheduled prompts or weighing times, especially in the absence of regular caregiver support. Taken together, these accounts suggest that advanced age, lower digital confidence, and limited support networks were important contextual factors that contributed to early disengagement from the intervention.

As shown in Table 3, the participating nurses were predominantly women, with a mean age of 40 (SD 6.1) years. A total of 65% (13/20) held clinical nurse positions, and, on average, they had been working full-time in home care for approximately 8 years.

**Table 3:** Profile of the participating nurses (n=20).

Characteristic		Value
Female, n (%)		19 (95)
Age (years), mean (SD)		40 (6.1)
**Job position, n (%)**		
	Registered nurse	7 (35)
	Clinical nurse	13 (65)
Average experience in home care (years), mean (SD)		8 (2.1)
Full-time job, n (%)		20 (100)

### Technofunctional Experience With Home Telemonitoring

#### Overview

We apply here the 4 core elements of the PBA to interpret the technofunctional experiences of patients and caregivers as they interacted with the multidevice home telemonitoring intervention. As mentioned earlier, this framework allows us to examine how participants engaged with the technologies cognitively, emotionally, and behaviorally within their daily lives. Following this thematic analysis, we present device-specific accounts to illustrate how these broader experiential patterns manifested across the 4 digital tools used in the intervention. These accounts help contextualize the variability in usability, perceived burden, and emotional response linked to each device.

#### Contextual Tailoring

Participants’ ability to engage with the home telemonitoring program depended strongly on how well the devices aligned with their daily routines, physical capabilities, and living environments. Many participants described entering unfamiliar territory when first encountering the digital tools. Initial apprehension was common, with patients voicing fears of breaking the equipment or failing to use it correctly. As 1 patient shared, “At first, I was nervous. I didn’t want to mess anything up, but the more I used it, the more confident I got” (Patient #7). These early fears diminished with hands-on practice, clear instructions, and encouragement from caregivers, demonstrating the importance of tailoring onboarding to individual learning styles and contexts.

However, integration into daily life was more difficult for participants whose routines were less predictable or socially constrained. For example, patients living in senior residences often reported difficulty aligning measurement times with scheduled meals or medication routines:

Sometimes I would miss the time to fill out the questionnaire or take my medication because I was still in the cafeteria. It depended on the day.

Patient #6

These observations highlight how routines must be considered in designing and delivering home telemonitoring interventions.

Connectivity issues compounded the challenge of learning. Several participants, particularly those living in rural settings, reported problems with Bluetooth synchronization or weak cellular signals, which led to repeated prompts or lost data. As 1 patient explained: “When it glitched, I would lose patience. I eventually figured out how to restart the system myself, but at first, I was overwhelmed” (Patient #23). These experiences illustrate the iterative process of digital learning, where contextual barriers often dictate the pace and success of adaptation.

#### Emotional and Cognitive Responses to Technology

Participants expressed a wide range of emotional responses to the home telemonitoring program, ranging from reassurance and increased confidence to frustration, fatigue, and anxiety. Many felt reassured knowing their health data were monitored in real time by a clinical team, “Even if she [nurse] wasn’t here, I felt like she was looking after me every day,” said one patient (Patient #11). This sense of remote presence contributed to perceived security, especially for those living alone.

Others, however, experienced anxiety, irritation, or emotional fatigue. This was especially true when dealing with repetitive tasks, frequent prompts, or nighttime alerts. As 1 patient remarked:

It felt useless to me, because it’s always the same questions… and the same answers. So, I eventually stopped responding to the robot.

Patient #1

A nurse confirmed these frustrations:

Some of my patients even mentioned being woken up repeatedly by the tablet’s sound alerts, which was experienced as a source of frustration.

Nurse #17

When feedback loops were missing, the technology was often perceived as intrusive or indifferent, diminishing its credibility and value.

#### Perceived Relevance and Burden of Technology Use

Participants evaluated the usefulness of the telemonitoring system in relation to the effort required to use it, and their experiences varied substantially across the different devices. The smartwatch, which required minimal user input, was generally well received. “I liked that I didn’t have to do much. But I never really knew what it was tracking or if it was working” (Patient #10). In contrast, the connected scale, though simple to use, lost value when readings were not acknowledged: “I weighed myself every morning, but it didn’t seem to matter. No one ever mentioned the numbers” (Patient #5). For its part, the voice-activated tablet received mixed reviews. While some appreciated the reminders, others struggled with the interface. “It would talk to me, but I couldn’t always hear it or figure out how to answer. It made me nervous” (Patient #21).

These examples suggest that bundling multiple devices under a single intervention may obscure the highly variable user experiences across technologies. Emotional and functional reactions depended not only on individual digital skills but also on how well each device fit into the participant’s everyday life.

#### Autonomy, Control, and Iterative Engagement

Over time, some participants developed greater confidence and autonomy in using the devices, whereas others experienced persistent difficulties that led to disengagement. For some participants, learning to troubleshoot devices fostered a sense of control and accomplishment. “After a while, I knew how to fix it myself. That made me feel more in control” (Patient #17). Others, however, experienced persistent problems that eroded their confidence. “I didn’t know what was wrong with the xPill, so I stopped using it. It was too stressful” (Patient #12). This divergence in experience illustrates the need for systems to support users not only during onboarding but throughout their engagement with technological devices.

Among participants who withdrew early, emotional and cognitive overload were frequent themes. “I just knew I wasn’t going to be able to manage it. It’s too much stress for someone like me” (Patient #20). In some cases, caregiver frustration contributed to early dropout: “My husband got frustrated with the machine after the second week. It was just easier to go back to the usual way” (Caregiver #4). These narratives point to the importance of iterative and adaptive support strategies to retain engagement over time.

### Relational and Experience Aspects of Remote Care

#### Perceived Professional Presence at a Distance

Many participants reported that the home telemonitoring program created a sense of ongoing clinical presence, even when direct communication with nurses was limited. This theme aligns with the person-centered processes domain of the PCP framework, particularly the dimension of sympathetic presence. Many participants described feeling reassured by the knowledge that their health status was being monitored, even in the absence of direct, face-to-face contact. One patient expressed this sense of invisible support: “Even if she [nurse] wasn’t here, I felt like she was looking after me every day” (Patient #11).

Nurses also made intentional efforts to convey a sense of presence despite the absence of face-to-face interactions. Several described how, without visual cues, they learned to attune more closely to patients’ vocal tone and inflection. As 1 nurse noted, “You have to ‘read’ the patient’s tone of voice… I could tell when someone wasn’t doing well just by how they said hello” (Nurse #2). To maintain an empathetic connection, some nurses also relied on open-ended questions and lengthier conversations. One nurse explained, “When I didn’t see their body language, I compensated by asking more open-ended questions” (Nurse #4).

These accounts illustrate that while digital devices introduce new barriers to physical proximity, person-centered care practices can still be upheld through deliberate and thoughtful communication strategies.

#### Shared Decision-Making and Tailored Interactions

Participants differed in the extent to which they wished to be involved in decisions related to the home telemonitoring program and their care. Shared decision-making represents a critical process within McCance and McCormack’s [11] framework. Participants’ experiences varied. Some felt genuinely involved when nurses contacted them to discuss their health status or device readings. One patient recounted: “She would call and ask how I felt before deciding anything. It felt personal” (Patient #5). Similarly, another participant explained how their input was acknowledged: “She asked me if it was okay to continue using the smart xPill since it was giving me trouble. I appreciated that” (Patient #9).

Others, however, preferred a more passive role and deferred entirely to clinical expertise. As 1 participant explained: “I just followed what they said. I trusted them” (Patient #13). A second participant echoed this sentiment: “I’m not the kind to ask questions. If the nurse says it’s good, I go with that” (Patient #18).

These differing accounts underscore the importance of communication strategies that are responsive to individual preferences for autonomy and involvement. While some users welcomed collaborative decision-making, others derived comfort from trusting clinical judgment, highlighting the need for flexibility in how shared decision-making is operationalized in remote care settings.

#### Emotional Reassurance and Well-Being

For many patients and caregivers, the intervention generated a sense of reassurance by signaling that clinical professionals were overseeing the patient’s condition. This theme maps onto the person-centered outcomes domain of McCance and McCormack’s [11] framework, specifically the importance of emotional well-being and reassurance. Several participants expressed comfort in knowing they were being watched over, even in the absence of direct communication. One patient put it simply: “It was reassuring, knowing someone was checking in, even if we didn’t talk every day” (Patient #1). Another noted: “It was nice to know someone was checking my data every day. Even if we didn’t talk, I felt watched over in a good way” (Patient #8). In short, this quiet sense of presence, mediated through the smart devices, was a recurring source of comfort for many.

Informal caregivers also shared this emotional benefit. Knowing that clinical professionals were monitoring their loved ones provided peace of mind and alleviated the perceived burden of constant vigilance. As 1 caregiver explained: “I knew that if something went wrong, the nurse would see the data and call. That took a weight off my shoulders” (Caregiver #4).

#### Limitations of Remote Engagement

Despite these benefits, participants also recognized important limits to digitally mediated care, particularly when compared with in-person interactions. This aligns with the contextual sensitivity emphasized in McCance and McCormack’s [11] framework. Some users acknowledged that the technological interface, while functional, could not replace the warmth and nuance of in-person interaction. One patient captured this sentiment: “The technology was good, but it didn’t replace a nurse sitting next to you, talking face to face” (Patient #12). Nurses echoed this concern. Some reported emotional strain stemming from the lack of tangible presence and the uncertainty of whether patients felt genuinely supported. One nurse reflected: “Sometimes I wondered if they [patients] felt alone with the devices. It’s not easy to know if your presence is felt” (Nurse #3).

Yet, many nurses believed that their efforts to stay in contact, personalize their communication, and respond promptly still conveyed a sense of relational continuity. One nurse recounted positive feedback from patients: “Several told me, ‘It’s like you're still here, even if we don’t see you.’ That’s what we wanted” (Nurse #1).

These perspectives reinforce the idea that, while digital health technologies alter the medium of interaction, person-centered care can still be practiced and felt if thoughtfully adapted.

## Discussion

### Principal Results

Using the complementary lenses of the PBA and the PCP frameworks, the study findings highlight three central insights: (1) engagement with home telemonitoring technologies depends strongly on users’ routines, digital confidence, and perceived burden; (2) relational support from nurses plays a critical role in shaping how patients interpret and tolerate technological challenges; and (3) informal caregivers often act as key mediators of sustained engagement with the system.

From a technofunctional perspective, findings highlight the importance of designing home telemonitoring programs that are emotionally acceptable, contextually adaptable, and responsive to user needs and constraints. Participants’ engagement varied widely based on their digital literacy, cognitive capacity, comfort with wearable or interactive devices, and the fit of the system with their daily routines. While some developed confidence over time, especially when supported by informal caregivers, others experienced persistent difficulties, particularly when their routines were irregular or when onboarding was not sufficiently individualized.

Participants often reacted emotionally to the devices, reporting reassurance, frustration, or anxiety depending on their ability to interact successfully with a particular technology. Repetitive symptom questionnaires and poorly timed alerts contributed to emotional fatigue, especially when feedback was absent or unclear. These reactions were compounded when technical issues such as Bluetooth disconnections or connectivity failures disrupted user routines or sleep. In some cases, persistent problems led to disengagement or early withdrawal.

Importantly, the intervention did not impact all devices equally. The smartwatch and scale were generally well accepted due to their ease of use and alignment with daily habits. In contrast, the tablet and smart pill dispenser posed greater usability and cognitive challenges, particularly for participants with lower digital confidence or limited caregiver support. These device-specific variations underscore the need to avoid one-size-fits-all solutions and instead tailor devices to user profiles and contextual factors.

From a relational standpoint, participants often found the experience of being monitored reassuring, provided that follow-up from nurses was timely and personalized. Short, routine interactions (eg, phone calls or messages) reinforced the sense of being cared for, even at a distance. However, when follow-up was perceived as inconsistent or impersonal, patients reported feeling overlooked or abandoned. Nurses themselves reported challenges in maintaining relational presence without visual cues and in addressing technical issues beyond their clinical role.

Informal caregivers emerged as critical, if underrecognized, mediators of technology use and continuity. Their availability, digital literacy, and relationship to the patient shaped the sustainability of engagement. Caregivers often provided troubleshooting support and emotional reassurance, especially in cases in which users felt overwhelmed by the technology.

Lastly, while the PBA and PCP frameworks helped structure our analysis along technofunctional and relational dimensions, participants’ experiences often spanned both domains simultaneously. In practice, engagement with the telemonitoring system emerged from the interaction between how users experienced the devices and how they experienced the care relationships surrounding those devices.

As shown in Table 4, participants’ experiences with the telemonitoring intervention cannot be fully understood through a purely technological or purely relational lens. Instead, many of the themes identified in the analysis reflect the interaction between technofunctional engagement with the devices and the relational dynamics of care. For instance, the sense of reassurance reported by many patients did not stem solely from the monitoring devices themselves, but from the perception that nurses were actively reviewing the transmitted data and would intervene if necessary. Similarly, frustrations associated with repetitive tasks or technical difficulties were often amplified when feedback from the clinical team was perceived as limited or delayed. Conversely, timely communication and empathetic follow-up frequently mitigated negative reactions to the technology and encouraged sustained engagement. Taken together, these findings suggest that the effectiveness and acceptability of home telemonitoring programs depend not only on the usability of digital tools but also on the quality of relational support surrounding their use.

**Table 4:** Integration of technofunctional and relational dimensions of participants’ experiences with home telemonitoring.

Integrated theme	PBA^a^ framework (technofunctional lens)	PCP^b^ framework (relational lens)	Combined insight
Reassurance through monitoring	Users interpreted device monitoring as health surveillance	Nurses’ follow-up created a sense of presence	Reassurance emerged from the interaction between technology and relational care, not from the device alone
Burden of repeated tasks	Repetitive questionnaires and alerts created fatigue	Lack of feedback reduced perceived care responsiveness	Techno-functional burden was amplified when relational feedback was missing
Adaptation and confidence	Users developed autonomy through learning the devices	Supportive nurses reinforced confidence	Sustained engagement depended on both usability and relational encouragement
Disengagement and withdrawal	Technical frustration and cognitive overload	Limited caregiver support and relational reassurance	Withdrawal often resulted from combined technical and relational gaps

^a^PBA: Person-Based Approach.

^b^PCP: Person-Centered Practice.

The following section situates these findings within the broader literature on home telemonitoring and digitally mediated care.

### Comparison With Prior Work

Our findings reinforce and extend prior research on home telemonitoring in aging populations, particularly along 2 key dimensions: technofunctional engagement and relational and experiential aspects of care. On the functional side, earlier studies have emphasized the importance of usability, ease of integration, and access to technical support in facilitating telehealth adoption [19,20]. This study builds on this foundation by identifying additional factors that influence sustained engagement, including emotional burden, alignment with daily routines, and users’ capacity to adapt and troubleshoot technical issues—insights that closely reflect the principles of the PBA [10]. In particular, this framework highlights the importance of contextual tailoring, emotional acceptability, and perceived autonomy, all of which were evident in our participants’ experiences.

Our findings also confirm prior evidence that alert fatigue, lack of personalized feedback, and the perceived irrelevance of prompts can erode motivation over time [20,21]. More broadly, the literature on older adults and digital health underscores the importance of designing interventions that are emotionally acceptable and minimally burdensome, rather than merely functionally efficient [8].

Although Feroz and Ahmad [22] did not explicitly apply the PBA, their systematic review of usability factors in secure mobile health apps aligns with many of its core tenets, including the role of emotional responses to technology, the challenges posed by unfamiliar digital environments, and the need for robust support structures. Our findings complement theirs by illustrating how these dynamics unfold in a complex, real-world home telemonitoring context involving multiple devices and interdependent roles among patients, caregivers, and nurses. In doing so, this study extends person-centered usability principles beyond app-based systems to the broader ecosystem of digitally mediated chronic care.

Turning to the relational and experiential dimensions of care, our findings align with prior research showing that digital systems can sustain a sense of connection between patients and health care professionals when supported by consistent follow-up and empathetic communication [23,24]. At the same time, few studies have examined these dynamics simultaneously from the perspectives of patients, informal caregivers, and nurses. By triangulating insights across these groups, this study highlights that the perceived quality of care relationships depends not only on the presence of user-friendly technological devices but also on the continuity and sensitivity of communication, factors essential to maintaining therapeutic engagement at a distance.

Our results also resonate with those of Murphy et al [25], who used McCance and McCormack’s [11] framework to examine older adults’ experiences with integrated care. Both studies emphasize the central role of sympathetic presence, authentic engagement, and shared decision-making in shaping how care is experienced. While Murphy et al [25] focused on in-person multidisciplinary care, our findings suggest that these person-centered processes can also be preserved and meaningfully experienced within digitally mediated care contexts.

Finally, the variation observed in participants’ preferences for involvement in decision-making can be interpreted through Savoli et al’s [26] typology of patient self-management styles. In their study of patient portal use, Savoli et al [26] identified 3 profiles—autonomous, engaged, and reliant—based on how patients attribute responsibility for self-management and perceive the role of technology. Participants in this study who welcomed dialogue and shared decision-making resembled the engaged profile, whereas those who preferred to defer decisions to nurses reflected the reliant profile. These parallels highlight the importance of tailoring communication and support strategies to individual preferences for autonomy and relational engagement in home telemonitoring programs.

### Implication for Practice

Building on these findings, several practical implications emerge for the design and implementation of home telemonitoring programs. Drawing on the principles of the PBA and PCP framework, we emphasize the importance of designing interventions that are emotionally acceptable, contextually tailored, and responsive to the lived experiences of patients and their caregivers.

First, onboarding and ongoing support must be individualized. Older adults vary widely in their levels of digital literacy, cognitive capacity, and learning preferences. Training should therefore be incremental and reinforced through scheduled follow-ups, with clear, accessible materials to help users navigate common technical challenges. Providing personalized guidance during the adaptation phase can reduce anxiety and build early confidence in system use.

Second, home telemonitoring tools must accommodate diverse daily routines. Our results highlight the friction created when device prompts conflict with users’ eating habits, medication schedules, or lifestyle preferences. Data entry and alert schedules should be easily configurable by patients or caregivers to minimize disruption and support sustainable engagement. Third, relational continuity remains essential, even in digitally mediated care. Participants valued consistent, personalized interactions with nurses, and expressed distress when follow-up felt irregular or impersonal. Assigning a dedicated referent nurse or implementing minimal standards for proactive communication may help reinforce the therapeutic relationship and enhance users’ sense of being seen and supported.

Fourth, regular feedback to patients is crucial for motivation. The absence of acknowledgment for submitted data diminished the perceived value of system use. Even brief, automated messages (eg, “Your data looks stable today”) can reinforce engagement, provide emotional reassurance, and maintain participation over time. Fifth, interfaces should allow for user-controlled modes of interaction. While some patients appreciated detailed access to their health data, others preferred to limit their exposure to potentially anxiety-inducing information. Home telemonitoring interventions should offer flexible configurations to respect these differences and promote a sense of control.

Sixth, caregivers require tailored onboarding and communication strategies. Their ability to support patients often depended on their understanding of how the technology worked and their preferred communication channel (eg, SMS, phone, or app). Dedicated support and training for caregivers, including technical troubleshooting and role clarification, can strengthen the triad of care among patients, caregivers, and clinicians. Seventh, technical reliability is foundational, especially in rural or low-connectivity environments. Participants reported repeated frustrations linked to connectivity failures, alerts triggered by errors, and synchronization issues. Hybrid network strategies (eg, Wi-Fi and cellular backup), intuitive error messaging, and simple troubleshooting instructions are critical to sustaining trust in the system.

Eighth, integration with existing clinical workflows and community resources must be prioritized. The effectiveness of tools like the smart xPill dispenser depends on accurate and updated medication profiles, which in turn require coordination with community pharmacists. Home telemonitoring programs should not operate in isolation, but rather as part of a broader, interprofessional care ecosystem.

Finally, while many participants reported positive experiences, it is important to acknowledge that frustration, emotional fatigue, and technical difficulties were common and, for some, led to early withdrawal. These negative experiences were often exacerbated by repetitive prompts, lack of real-time feedback, or device malfunction, especially in the absence of caregiver support. For several patients, the cognitive and emotional effort required to engage with the devices outweighed the perceived benefits. These findings underscore a critical tension in distant care. Even well-designed interventions can generate unintended burdens that erode engagement over time. As such, scaling similar home telemonitoring programs without addressing these experiential barriers may exacerbate disparities in access, increase dropout rates, and strain frontline support systems. Future implementations should therefore prioritize sustained user support, adaptive personalization, and continuous feedback mechanisms to mitigate these risks.

### Study Limitations

Three main limitations should be considered when interpreting the findings of this study. First, the transferability of our results is constrained by the study’s geographic scope and sample size. Data were collected from 3 health care organizations located within a single Canadian province, and only 34 patients and their caregivers completed the full duration of the pilot program. Although the participating sites were selected to represent rural, semiurban, and urban contexts, the relatively small number of participants and the contextual specificity of the intervention limit the extent to which the findings can be generalized to broader populations or settings.

At the same time, the goal of this qualitative study is not statistical generalization but analytical transferability. By providing detailed descriptions of the intervention, participant characteristics, and care context, we aim to enable readers to assess the extent to which the patterns identified in this study—such as the interplay between technological usability, caregiver support, and relational continuity with clinicians—may resonate with similar home telemonitoring initiatives in other health care environments.

The transferability of our findings to contexts outside Canada should also be interpreted with caution. The home telemonitoring program was implemented within the publicly funded Canadian health care system, where home care services, nursing roles, and digital health initiatives may be organized differently than in other countries. For instance, the involvement of home care nurses in reviewing patient data and conducting follow-up reflects organizational features that may not be present in all health systems. Nevertheless, several of the dynamics identified in this study, including the influence of digital literacy, caregiver involvement, technological burden, and the relational aspects of remote care, have been reported in home telemonitoring initiatives in other settings, suggesting that these findings may still offer useful insights for similar programs implemented in different health care contexts.

Second, the study may be subject to selection bias. Indeed, many patients agreed to participate based on a strong preexisting relationship with their home care nurse, suggesting that trust in the health care professional played a central role in motivating enrollment. Others were motivated by altruistic values such as a desire to contribute to research or “give back” to the health system. These motivations, while valuable, may have skewed the participant sample toward individuals more likely to engage with the intervention or tolerate its demands, potentially underrepresenting the perspectives of those with lower motivation, weaker support networks, or more limited digital literacy.

Third, although we used a rigorous approach to directed content analysis [19], the inherently interpretive nature of qualitative research carries the risk of subjectivity in coding and theme development. Even though coding was performed rigorously, the analytical process was inevitably shaped by our prior assumptions, research objectives, and theoretical framing. The risk of interpretive bias is especially salient given that our analysis was guided by 2 preexisting conceptual frameworks, which, while helpful in structuring the analysis, may have constrained the emergence of unexpected insights. We sought to mitigate this risk by maintaining an iterative coding process that remained open to experiential elements not fully captured by the frameworks. Divergent accounts, including frustration, disengagement, and early withdrawal from the program, were examined alongside more positive experiences to ensure that the analysis reflected the diversity of participants’ perspectives.

### Conclusions

Home telemonitoring programs that involve multiple digital devices can empower older adults and their caregivers, but only when they are flexibly integrated into daily routines, emotionally acceptable, and consistently supported both technically and relationally. The PBA provided a valuable framework for interpreting how users engaged with the intervention across functional, emotional, and contextual dimensions. It emphasized the importance of personalization, perceived relevance, and iterative adaptation in shaping technology use over time. Complementing this, the PCP framework helped illuminate how participants experienced presence, shared decision-making, and relational continuity, even in the absence of face-to-face contact. This framework foregrounded the relational and emotional texture of care, drawing attention to the processes through which users felt acknowledged, supported, and empowered.

By combining these 2 lenses, this study captures both the tangible functionality and the intangible relational dynamics of digitally mediated care. Together, they underscore the need for patient- and caregiver-centered design principles, sustained interpersonal engagement, and robust interprofessional coordination. Evaluating home telemonitoring interventions through both technofunctional and relational perspectives offers a fuller understanding of how such programs are lived and how they can be more effectively implemented at scale.

## Data Availability

The datasets generated or analyzed during this study are available from the corresponding author on reasonable request.

## Appendix

Multimedia Appendix 1 Interview guides.

## Abbreviations

CISSS: Centre intégré de santé et de services sociaux
HF: heart failure
PBA: Person-Based Approach
PCP: Person-Centered Practice

## References

[1] Goyal P, Maurer MS, Roh J (2024). Aging in heart failure: embracing biology over chronology: JACC family series. JACC Heart Fail.

[2] Liu G, Nguyen NQH, Wong KE, Agarwal SK, Boerwinkle E, Chang PP (2024). Metabolomic association and risk prediction with heart failure in older adults. Circ Heart Fail.

[3] Jaana M, Sherrard H, Paré G (2019). A prospective evaluation of telemonitoring use by seniors with chronic heart failure: adoption, self-care, and empowerment. Health Informatics J.

[4] Kitsiou S, Paré Guy, Jaana M (2015). Effects of home telemonitoring interventions on patients with chronic heart failure: an overview of systematic reviews. J Med Internet Res.

[5] Li L, Ringeval M, Wagner G, Paré G, Ozemek C, Kitsiou S (2025). Effectiveness of home-based cardiac rehabilitation interventions delivered via mHealth technologies: a systematic review and meta-analysis. Lancet Digit Health.

[6] Paré G, Moqadem K, Pineau G, St-Hilaire C (2010). Clinical effects of home telemonitoring in the context of diabetes, asthma, heart failure and hypertension: a systematic review. J Med Internet Res.

[7] Jaana M, Paré Guy (2020). Comparison of mobile health technology use for self-tracking between older adults and the general adult population in Canada: cross-sectional survey. JMIR Mhealth Uhealth.

[8] Yan M, Sun W, Tan C, Wu Y, Liu Y (2025). Analysis of factors influencing the willingness of Chinese older adults to use mHealth devices: nationwide cross-sectional survey study. J Med Internet Res.

[9] Castonguay A, Paré G, Moreault MP, Hardy MS, Ringeval M, Voyer P (2026). Implementation of a multidevice telemonitoring program for home-based nursing care in Quebec: qualitative report. JMIR Med Inform.

[10] Yardley L, Morrison L, Bradbury K, Muller I (2015). The person-based approach to intervention development: application to digital health-related behavior change interventions. J Med Internet Res.

[11] McCance T, McCormack B (2025). The person-centred nursing framework: a mid-range theory for nursing practice. J Res Nurs.

[12] Bate P, Robert G (2006). Experience-based design: from redesigning the system around the patient to co-designing services with the patient. Qual Saf Health Care.

[13] Voice activated tablet. Samsung.

[14] Smartwatch. Biobeat.

[15] Bluetooth-enabled weight scale. A&D Medical.

[16] xPill pro. Domedic.

[17] Voyer P (2017). L’examen clinique de l’aîné.

[18] Virtuose Technologies.

[19] Hsieh HF, Shannon SE (2005). Three approaches to qualitative content analysis. Qual Health Res.

[20] Greenhalgh T, Wherton J, Papoutsi C, Lynch J, Hughes G, A'Court C (2017). Beyond adoption: a new framework for theorizing and evaluating nonadoption, abandonment, and challenges to the scale-up, spread, and sustainability of health and care technologies. J Med Internet Res.

[21] Pols J (2012). Care at a Distance: On the Closeness of Technology.

[22] Feroz I, Ahmad N (2024). Systematic review of usability factors, models, and frameworks with blockchain integration for secure mobile health (mHealth) applications. Blockchain Healthc Today.

[23] Green CA, Polen MR, Janoff SL, Castleton DK, Wisdom JP, Vuckovic N (2008). Understanding how clinician-patient relationships and relational continuity of care affect recovery from serious mental illness: STARS study results. Psychiatr Rehabil J.

[24] Morris ME (2022). Enhancing relationships through technology: directions in parenting, caregiving, romantic partnerships, and clinical practice. Dialogues Clin Neurosci.

[25] Murphy S, Doyle M, Gallagher N (2023). “They talk to you like you’re a person”: validating McCormack and McCance’s person-centred practice framework through a qualitative study of older people’s experiences of person-centred integrated care. Int J Integr Care.

[26] Savoli A, Barki H, Pare G (2020). Examining how chronically ill patients? Reactions to and effective use of information technology can influence how well they self-manage their illness. MIS Quarterly.

[27] Leung TI, de Azevedo Cardoso T, Mavragani A, Eysenbach G (2023). Best practices for using AI tools as an author, peer reviewer, or editor. J Med Internet Res.

