# Supplementary material for: Experiences of Older Adults and Caregivers With Home Telemonitoring for Heart Failure in Canada: Qualitative Study
Source: JMIR Aging. 2026 Jun 16;9:e79797. doi: 10.2196/79797 (PMC13320013; doi:10.2196/79797)
Supplement: Multimedia Appendix 1 [file aging_v9i1e79797_app1.docx]

**Multimedia Appendix 1. Interview guides**

**Interview Guide – Patients**

*Introduction:*

- Can you briefly describe your experience participating in the telemonitoring program?

*Experience with the technology (PBA-informed):*

1. How did you feel when you first started using the devices?
2. Which devices were easiest or most difficult to use? Why?
3. How did using these devices fit into your daily routine?
4. Did you experience any frustrations or difficulties with the technology?
5. Did the system ever make you feel reassured or worried about your health?

*Perceived usefulness and burden (PBA-informed):*

1. In what ways, if any, did the system help you manage your health?
2. Were there moments when using the system felt burdensome or tiring?

*Relationship with the care team (PCP-informed):*

1. How did the monitoring system influence your relationship with the nurse?
2. Did you feel that someone was “watching over” your health? Can you explain?
3. Did the nurses involve you in decisions about your care or the use of the system?

*Overall experience:*

1. What aspects of the program worked particularly well for you? What aspects should be improved?

**Interview Guide – Nurses**

*Introduction:*

- Can you describe your experience supporting patients in this telemonitoring program?

*Experience with the technology (PBA-informed):*

1. How easy or difficult was it for patients to use the different devices?
2. What types of technical challenges did patients most frequently encounter?
3. How did these challenges affect patient engagement with the system?

*Integration into care practices:*

1. How did telemonitoring influence your daily clinical work?
2. Did the system help you detect potential health issues earlier?

*Relational aspects of care (PCP-informed):*

1. How did the telemonitoring system affect your relationship with patients?
2. Did it change how you communicated with them?
3. Were there situations where you felt the technology supported or hindered person-centred care?

*Patient engagement and outcomes:*

1. What factors seemed to influence whether patients continued using the system or withdrew?
2. What role did caregivers play in patient engagement?

*Reflections on the program:*

1. In your view, what were the main strengths of this telemonitoring program?
2. What changes would improve future implementations?

**Interview Guide – Informal caregivers**

*Introduction:*

- Can you briefly describe your role in supporting your relative during the telemonitoring program?

*Experience with the technology (PBA-informed):*

1. How did you first feel about your relative participating in the telemonitoring program?
2. How easy or difficult was it for your relative to use the devices? Did you personally help them use the system or troubleshoot problems? If so, how?

*Perceived usefulness and burden (PBA-informed):*

1. Did the system create additional responsibilities or stress for you as a caregiver?

*Relationship with the care team (PCP-informed):*

1. Did the telemonitoring program change how you interacted with nurses or healthcare professionals?

*Emotional reassurance and caregiving experience (PCP-informed):*

1. Did the system make you feel more reassured or confident about your relative’s care?

*Overall reflections:*

1. From your perspective, what worked well in this program?
